# Epistatic interactions promote persistence of NS3-Q80K in HCV infection by compensating for protein folding instability

**DOI:** 10.1016/j.jbc.2021.101031

**Published:** 2021-07-31

**Authors:** Georg Dultz, Sanjay K. Srikakulam, Michael Konetschnik, Tetsuro Shimakami, Nadezhda T. Doncheva, Julia Dietz, Christoph Sarrazin, Ricardo M. Biondi, Stefan Zeuzem, Robert Tampé, Olga V. Kalinina, Christoph Welsch

**Affiliations:** 1 Department of Internal Medicine 1, Goethe University Hospital Frankfurt, Frankfurt am Main, Germany; 2 Helmholtz Institute for Pharmaceutical Research Saarland (HIPS), Helmholtz Centre for Infection Research, Saarland University Campus, Saarbrücken, Germany; 3 Graduate School of Computer Science, Saarland University, Saarbrücken, Germany; 4 Interdisciplinary Graduate School of Natural Product Research, Saarland University, Saarbrücken, Germany; 5 Department of Gastroenterology, Kanazawa University Hospital, Kanazawa, Japan; 6 Novo Nordisk Foundation Center for Protein Research, University of Copenhagen, Copenhagen, Denmark; 7 Molecular Targeting, Instituto de Investigación en Biomedicina de Buenos Aires (IBioBA) - CONICET - Partner Institute of the Max Planck Society, Buenos Aires, Argentina; 8 University Center for Infectious Diseases, University Hospital Frankfurt, Frankfurt am Main, Germany; 9 Institute of Biochemistry, Biocenter, Goethe University Frankfurt, Frankfurt am Main, Germany; 10 Medical Faculty, Saarland University, Homburg, Germany; 11 Center for Bioinformatics, Saarland Informatics Campus, Saarbrücken, Germany

**Keywords:** hepatitis C virus, serine protease (NS3-4A), protein folding, protein evolution, viral fitness, immune escape, resistance mutation, CTL, cytotoxic T lymphocyte, DAA, direct-acting antiviral, FC, fold change, GLuc, Gaussia luciferase, HCV, hepatitis C virus, MD, molecular dynamics, PI, protease inhibitor

## Abstract

The Q80K polymorphism in the NS3-4A protease of the hepatitis C virus is associated with treatment failure of direct-acting antiviral agents. This polymorphism is highly prevalent in genotype 1a infections and stably transmitted between hosts. Here, we investigated the underlying molecular mechanisms of evolutionarily conserved coevolving amino acids in NS3-Q80K and revealed potential implications of epistatic interactions in immune escape and variants persistence. Using purified protein, we characterized the impact of epistatic amino acid substitutions on the physicochemical properties and peptide cleavage kinetics of the NS3-Q80K protease. We found that Q80K destabilized the protease protein fold (*p* < 0.0001). Although NS3-Q80K showed reduced peptide substrate turnover (*p* < 0.0002), replicative fitness in an H77S.3 cell culture model of infection was not significantly inferior to the WT virus. Epistatic substitutions at residues 91 and 174 in NS3-Q80K stabilized the protein fold (*p* < 0.0001) and leveraged the WT protease stability. However, changes in protease stability inversely correlated with enzymatic activity. In infectious cell culture, these secondary substitutions were not associated with a gain of replicative fitness in NS3-Q80K variants. Using molecular dynamics, we observed that the total number of residue contacts in NS3-Q80K mutants correlated with protein folding stability. Changes in the number of contacts reflected the compensatory effect on protein folding instability by epistatic substitutions. In summary, epistatic substitutions in NS3-Q80K contribute to viral fitness by mechanisms not directly related to RNA replication. By compensating for protein-folding instability, epistatic interactions likely protect NS3-Q80K variants from immune cell recognition.

The polymorphism Q80K in the NS3-4A protease of hepatitis C virus (HCV) dates back to a single substitution event around the 1940s in the United States and is associated with reduced treatment response on direct-acting antivirals (DAAs) (1, 2, 3, 4). Depending on the geographic region, NS3-Q80K is found in up to 48% of untreated genotype 1a–infected patients, whereas it is rarely seen in genotype 1b infection (approximately 0.5%) (5). Although the resistance level for NS3-Q80K against protease inhibitors (PIs) was determined to be rather low, only approximately 10-fold change (FC) (6), its clinical consequences are striking. In large interferon-based clinical trials of the PI simeprevir, Q80K was highly associated with genotype 1a–treatment failure leading to response rates not superior to pegylated interferon-α plus ribavirin (7). Dietz *et al.* (8) reported an increase from 35% to 56% of Q80K as a resistance-associated amino acid substitution in genotype 1a for simeprevir in combination with the nucleotide NS5B inhibitor sofosbuvir. A similar although weaker increase in Q80K is reported for the 3D regimen (PrOD), a triple combination comprising the PI paritaprevir/ritonavir, the NS5A inhibitor ombitasvir, and the non-nucleoside NS5B inhibitor dasabuvir. Notably, 41% of individuals with failure on 3D and NS5A-RASs harbored NS3-Q80K, which is a higher prevalence compared with 35% in DAA-naïve patients from this study (8). The long phylogenetic history of NS3-Q80K, however, is in sharp contrast with those of other drug resistance mutations and indicates that it does not arise as a response to drug selection (1).

By reconstruction of the evolutionary history of the Q80K polymorphism, epistatic residue interactions are identified descendent from a single NS3-Q80K lineage that may compensate stabilizing or functional properties of the protease structure, S174N and A91S/T^1^. Epistasis is widely recognized as a key phenomenon that drives the dynamics of evolution. It occurs when the combined effect of two or more mutations differs from the sum of their individual effects and arguably reflects molecular interactions that affect the function of a protein (9, 10). Characterizing high-order epistasis between three or more mutations can unveil hidden intramolecular interaction networks that underlie essential molecular mechanisms related to protein functions and their evolution (11). Multidomain proteins such as the NS3 protein of HCV are complex molecular machines with their biophysical and functional properties not simply additive. The N-terminal protease of NS3 is characterized by a bilobal structure that requires the structural complementation by the NS4A peptide derived from the viral polyprotein (12). By proteolytically processing nonstructural proteins from the viral polyprotein, the protease is involved in RNA synthesis (13). In addition, the capacity to cleave and inactivate the mitochondrial antiviral-signaling protein MAVS (also called Cardif, VISA, and IPS-1) in the retinoic acid–inducible gene I signaling pathway is a cardinal feature of NS3-4A, by which HCV blocks induction of interferon-β (14, 15, 16, 17, 18). The NS4A peptide intercalates into the protease structure to support proper protein folding and full enzymatic activity (13). A linker connects the protease domain to a C-terminal RNA helicase with both domains being highly interdependent (19, 20). By interference with the NS3 helicase domain, the protease modulates infectious virus particle assembly (20, 21). The NS3-4A protease is a prime drug target against HCV (20). Its structure and function is conserved along the family of flaviviridae and is a target for new drugs against emerging human pathogens, such as dengue, West Nile, or yellow fever virus (22). RASs in NS3-4A of HCV frequently lead to considerable fitness costs that for most mutations result from reductions in RNA replication capacity and for some in impairment in their ability to produce infectious virus (23).

In the present study, we show that epistatic interactions in the NS3-4A protease stabilize the protein fold of NS3-Q80K variants and leverage protein stability to the WT level. In agreement with the overall protein stability of NS3-Q80K, we find the total number of noncovalent residue interactions increasing upon amino acid substitution at epistatic interaction sites, although some of these residues do not immediately interact with residues at position 80. Changes in protein stability because of epistatic interactions showed a trade-off against protease enzymatic activity and replicative fitness deficits in infectious cell culture. The finding that epistatic residue interactions are not associated with a gain-of-function phenotype in replicative fitness of NS3-Q80K variants appears surprising, given the high prevalence of these substitutions in genotype 1a. Our data suggest that immune-escape mechanisms could contribute to the overall fitness of NS3-Q80K variants *in vivo*.

## Results

### Rational approach

**Figure 1:**
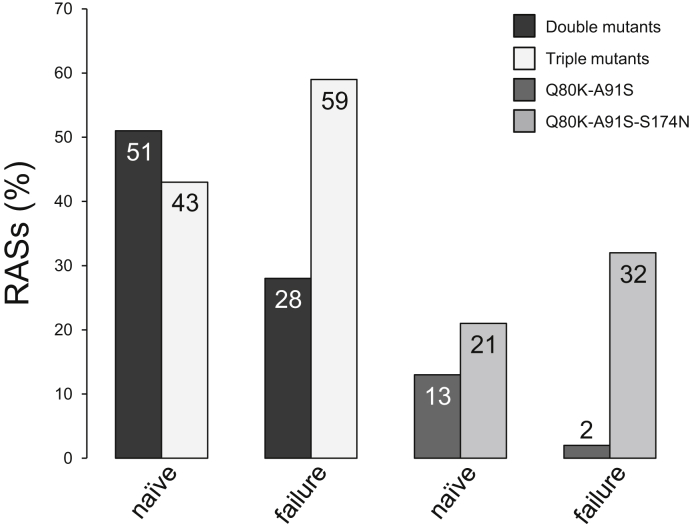
**Prevalence of epistatic secondary substitutions in DAA-naïveand DAA-experienced patients harboring NS3-Q80K.** Comparison of mutant patterns in patients with genotype 1a HCV infection harboring NS3-Q80K between the European HCV database (24), euHCVdb (DAA naïve, n = 100), and DAA-experienced patients harboring NS3-Q80K from the Frankfurt Resistance Database (upon DAA failure, n = 41). Data shown represent percentages (%) of patients from which the respective variants were isolated as major variants before DAA treatment or upon treatment failure. DAA, direct-acting antiviral; HCV, hepatitis C virus.

We explored epistasis and mutual dependence of distinct sites in the NS3-4A protease structure as a possible mechanism to explain the prevalence of NS3-Q80K in genotype 1a HCV infection. The mutant patterns are selected based on an article by McCloskey *et al.* (1) We analyzed sequence data of genotype 1a–infected patients with NS3-Q80K for epistatic secondary substitutions at residue 91 and/or 174 based on sequence information from DAA-experienced patients from the Frankfurt Resistance Database and sequence information from DAA-naïve patients from the European HCV database (24) (see supporting information). In DAA-experienced patients, we found triple mutant patterns, that is Q80K-A91S-S174N and Q80K-A91T-S174N, selected over double mutant patterns, Q80K-A91S/T and Q80K-S174N (Fig. 1). Subsequently, we characterized the impact of epistatic residue interactions on the physicochemical properties of the protease by synthetic biology using purified protein and complementary bioinformatics approaches. We then used a cell culture model of HCV infection to assess implications of epistatic residue interactions in NS3-4A for the replicative fitness of NS3-Q80K variants.

### Epistatic secondary substitutions in NS3-Q80K stabilize the protease protein fold

**Figure 2:**
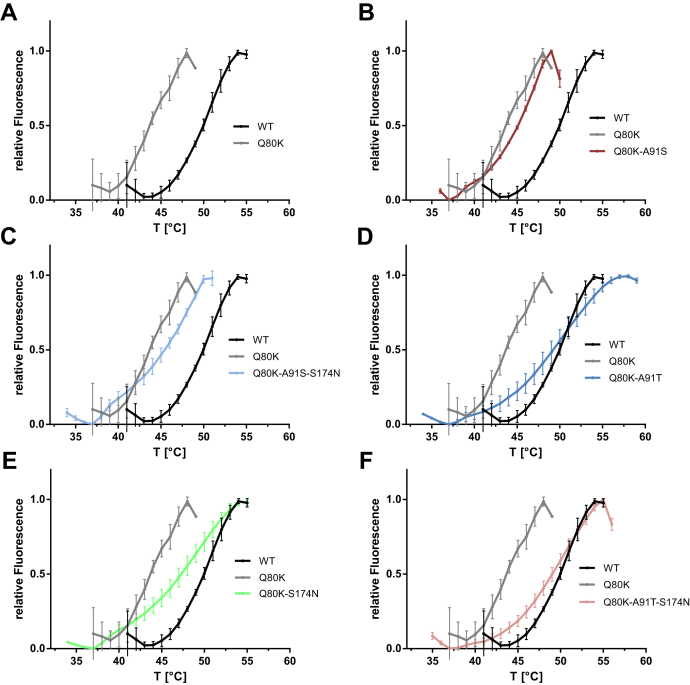
**Thermal shift of the NS3-4A protease harboring NS3-Q80K and epistatic amino acid substitutions.** Data from real-time thermal stability assay using Sypro Orange, a temperature-stable fluorophore that exhibit enhanced fluorescence upon interacting with unfolded proteins. *A*, thermal stability of the WT protease and the NS3-Q80K mutant, as well as (*B*–*F*), mutants harboring epistatic secondary substitutions assessed under increasing incubation temperatures. Graphs showing impact of mutations on protease protein unfolding patterns characterized by fluorescence emission curves.

**Figure 3:**
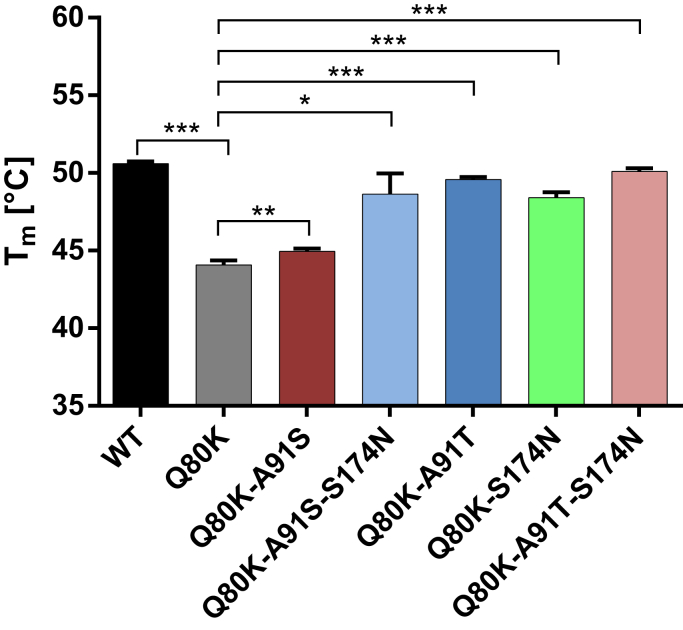
**Impact of NS3-Q80K and epistatic amino acid substitutions on the protease protein fold.***T*_m_ from purified NS3-4A protease WT and mutants are determined by fitting the sigmoidal melt curve as shown in Figure 2 to the Boltzmann equation. Error bars represent the mean ± SD from at least three independent experiments; ∗*p* ≤ 0.05; ∗∗*p* ≤ 0.01; and ∗∗∗*p* ≤ 0.001; by two-sided *t* test.

To characterize the impact of NS3-Q80K and its epistatic amino acid substitutions S174N and A91S/T on the protease protein fold, we expressed and purified the NS3-4A WT fusion and different mutant forms (see Experimental procedures section). We tested if NS3-Q80K ± S174N and/or A91S/T had an effect on the protease stability (Figs. 2 and 3 and Table 1). The impact of mutants on protein folding was determined by a thermal shift assay, where protein denaturation at increasing temperatures is revealed by increased fluorescence (25). The NS3-4A protease WT was stable to temperature with a half maximal denaturation, *T*_m_, at 50.6 °C (Figs. 2 and 3), whereas the *T*_m_ of NS3-Q80K revealed a significant destabilizing effect on protein stability, *T*_m_ at 44.1 °C (*p* < 0.0001) (Figs. 2 and 3). We find that all epistatic amino acid substitutions shifted the denaturation curve to higher temperatures and stabilized the protease fold of the respective expressed NS3-Q80K mutant proteins (Fig. 2). All mutants, but Q80K-A91S, showed compensation in protein stability that equals the level of the WT protein (Fig. 3). Notably, the double mutants Q80K-A91T (*T*_m_ at 49.6 °C) (*p* < 0.0001) and Q80K-S174N (*T*_m_ at 48.4 °C) (*p* < 0.0001) and both triple mutants, Q80K-A91S-S174N (*T*_m_ at 48.6 °C) (*p* = 0.018) and Q80K-A91T-S174N (*T*_m_ at 50.1 °C) (*p* < 0.0001), showed no difference in protein stability with the WT protease (Fig. 3 and Table 1). Only weak stabilization of the protease fold (*p* < 0.04) was observed for Q80K-A91S (Fig. 2). Among all mutant patterns, the melting curve of the triple mutant Q80K-A91T-S174N closest resembles the curve of the WT protease protein (Fig. 2). Interestingly, the denaturation curves do not show the expected denaturation curves parallel to the denaturation curve of the WT. Instead, the Q80K mutation shows a clear deviation from the expected curve and reproducibly possesses a shoulder during denaturation (Fig. 2*A*). The result suggests that NS3-Q80K differentially affect the two subdomains of the protease that build the bilobal structure of the protein. In solution, upon heating, the protease subdomain comprising the Q80K mutation may denature separately. Notably, epistatic secondary substitutions that compensate Q80K also show a clear shoulder (Fig. 2, *B*–*F*) and a denaturation curve that does not parallel the denaturation of the WT protein. It appears then that NS3-Q80K not only destabilizes the protein but also may affect the communication between the two lobes of the protease. Since the active site of the protease is located in the region corresponding to the connection between the two lobes of the protease, the NS3-Q80K mutation and the epistatic secondary substitutions may affect the protease catalytic activity.

**Table 1:** Enzyme kinetic constants and protease protein *T*_m_s for the WT protease and NS3-Q80K variants with epistatic amino acid substitutions

Variant	Enzyme kinetics		Variant	*T*_m_ (°C)
	*K*_*M*_ (μM)	*k*_cat_ (/min)		
WT (Q80)	2.71 ± 0.31	5.46 ± 0.26	WT (Q80)	50.6 ± 0.15
Q80K	1.44 ± 0.24	1.8 ± 0.1	Q80K	44.1 ± 0.29
Q80K-A91S	0.56 ± 0.06	1.56 ± 0.06	Q80K-A91S	45.0 ± 0.19
Q80K-A91T	0.48 ± 0.13	0.4 ± 0.03	Q80K-A91T	49.6 ± 0.16
Q80K-S174N	0.14 ± 0.03	0.48 ± 0.02	Q80K-S174N	48.4 ± 0.37
Q80K-A91S-S174N	8.16 ± 4.62	0.13 ± 0.04	Q80K-A91S-S174N	48.6 ± 1.33
Q80K-A91T-S174N	0.67 ± 0.09	2.41 ± 0.11	Q80K-A91T-S174N	50.1 ± 0.21

Kinetic constants and melting temperatures obtained by FRET-based protease and thermal shift assay, respectively. Data shown represent the mean ± SD from at least three independent experiments.

### Effect of mutations on the catalytic activity of the protease

**Figure 4:**
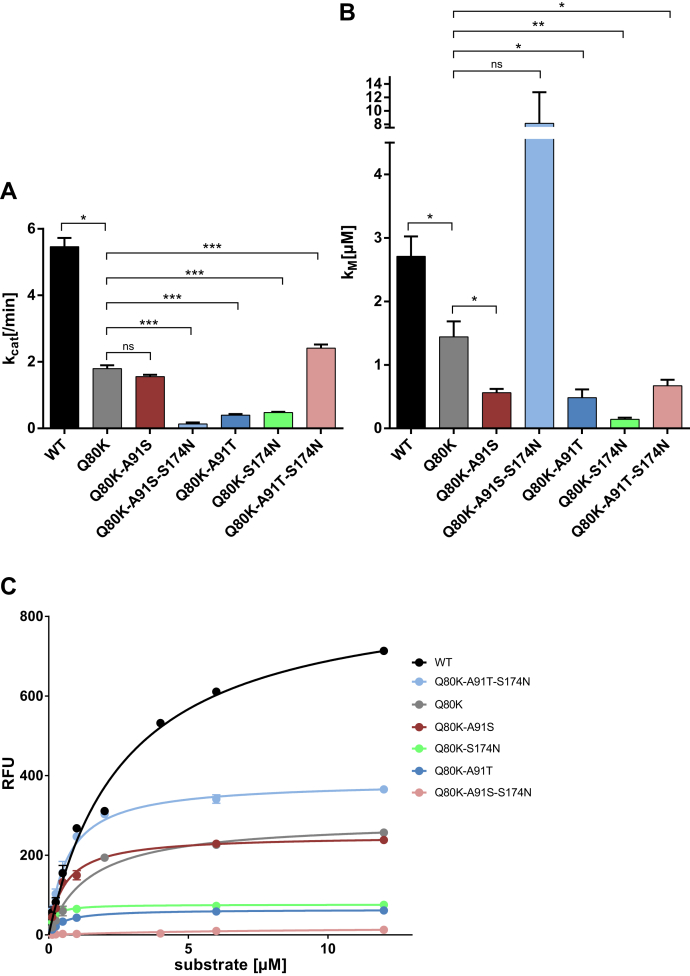
**Impact of NS3-Q80K and epistatic amino acid substitutions on the protease enzymatic function and reaction velocity.** Reaction velocity and Michaelis–Menten kinetics as assessed from purified protein of WT protease and NS3-Q80K mutants and the natural polyprotein substrate NS4A/4B. *A*, reaction constants *k*_cat_ and (*B*) *K*_*M*_ calculated after nonlinear regression curve fitting. Error bars represent the mean ± SD from at least three independent experiments; ∗*p* ≤ 0.05; ∗∗*p* ≤ 0.01; and ∗∗∗*p* ≤ 0.001; by two-sided *t* test. *C*, kinetic progress curves with reaction velocities (RFU, relative fluorescence unit) for the NS3-4A protease and the natural polyprotein substrate NS4A/4B as assessed from purified protein of WT protease and NS3-Q80K mutants.

We considered it likely that epistatic amino acid substitutions in NS3-4A might rescue replicative fitness deficits from NS3-Q80K and potentially explain the high prevalence of this polymorphism. To assess a potential direct impact of the stabilized protein fold on the protease catalytic activity, we analyzed the enzymatic activity using an *in vitro* FRET-based assay that measures the cleavage of a viral polyprotein substrate derived from the viral NS4A/4B polyprotein cleavage site (26). In this assay, fluorescence is released when the protease recognizes and cleaves the FRET substrate (see Experimental procedures section). We tested the kinetic properties of the NS3-4A protease harboring NS3-Q80K ± S174N and/or A91S/T. We determined the amount of substrate needed to obtain half of the protease maximum rate of reaction (*K*_*M*_) and substrate turnover rates (*k*_cat_). The protease harboring NS3-Q80K exhibited a lower substrate turnover rate (*p* = 0.0002; FC = 0.33) and lower Michaelis–Menten constant, *K*_*M*_, than the NS3-Q80 protease WT (*p* = 0.03; FC = 0.53). Except for the Q80K-A91S-S174N triple mutant (with 8.16 μM), *K*_*M*_, in all mutant patterns tested, was significantly lower than in NS3-Q80K and only varied in rather narrow ranges between 0.14 and 0.67 μM (Fig. 4 and Table 1). Roughly, the low *K*_*M*_ indicates higher substrate affinity in the NS3-Q80K protease comprising epistatic amino acid substitutions than in the NS3-Q80 protease WT or NS3-Q80K variant without epistatic secondary substitutions. The protease comprising epistatic secondary substitutions likely requires a lower concentration of peptide substrate to achieve the maximum rate of reaction. However, this was not paralleled by an increase in the substrate turnover rates, *k*_cat_, which were significantly inferior to WT for all mutant patterns tested. Only the Q80K-A91S double mutant (equal level; *p* = 0.1) and the Q80K-A91S-S174N triple mutant (increased substrate turnover; *p* = 0.015) showed substrate turnover that leveled up with NS3-Q80K. Noteworthy, in full-length NS3, the helicase domain can stimulate protease activity (19, 20). In our enzymatic activity assay, however, the expressed protease constructs lack the NS3 helicase domain (27) and potentially do not capture indirect mechanisms related to the interaction of the protease and helicase domain of NS3.

Collectively, our biochemistry data show that Q80K clearly decreases the catalytic turnover of the NS3-4A protease, whereas epistatic secondary substitutions do not compensate on this. The *in vitro* catalytic turnover of a cellular protease, however, may not reflect the efficiency by which it accomplishes its physiological role, since the concentration of protease substrates *in vivo* may be limiting. Hence, we conclude that the catalytic turnover may not be limiting viral replication. Alternatively, the destabilization on the Q80K lobe of the protease, as suggested by the temperature stability experiments, could render a compensating effect. We speculated that destabilization of protein regions potentially enable optimized interaction with protease physiological substrates. In line with this idea, the decreased *K*_*M*_ toward the polyprotein peptide substrate (Fig. 4*B*) already suggests that peptide substrates may bind with higher affinity to NS3-4A in the context of the destabilized protein.

### Protein dynamics and inter-residue contacts explain protease fold stabilization in NS3-Q80K harboring epistatic secondary substitutions

**Figure 5:**
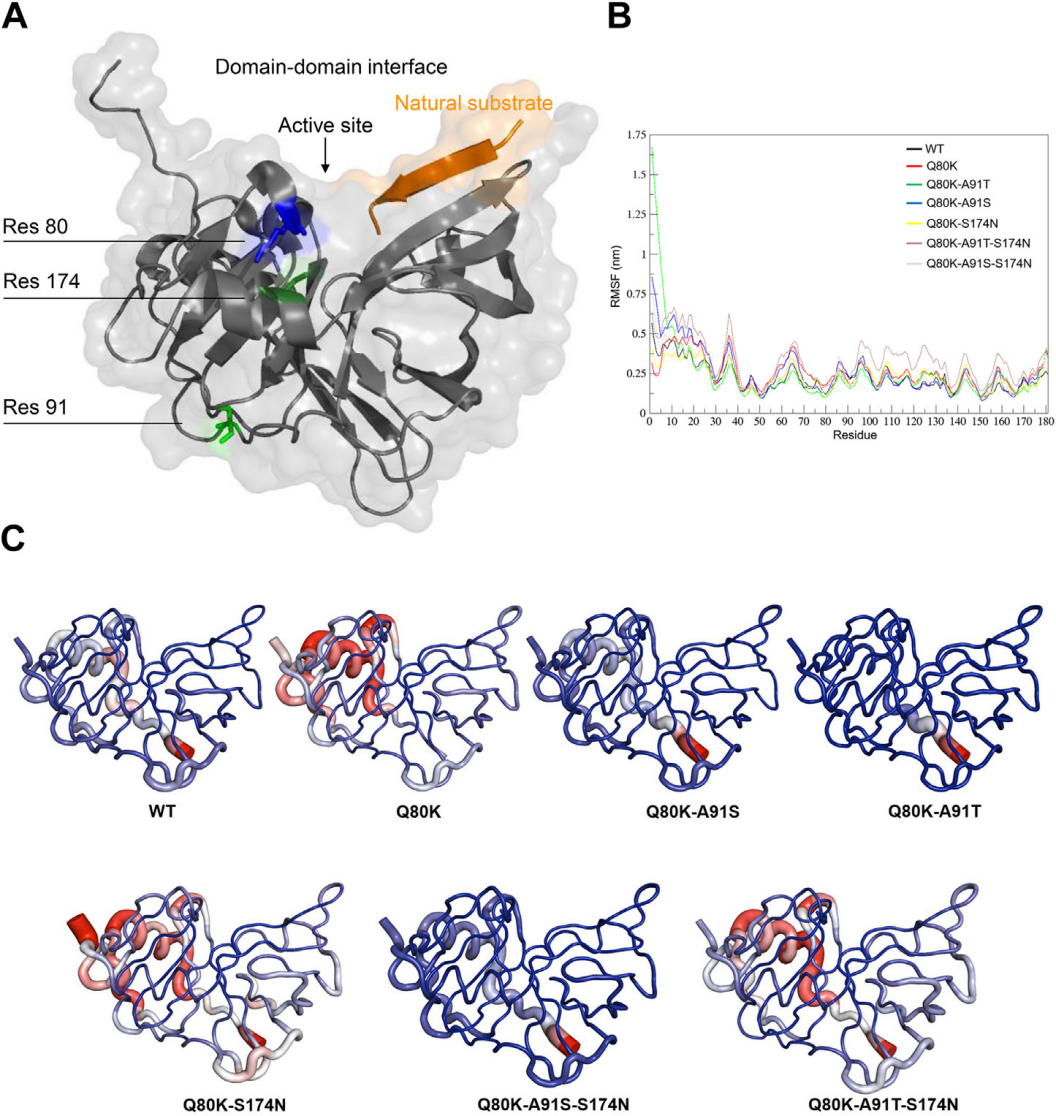
**Protease fold and protein flexibility estimated by root mean square fluctuations (RMSFs).***A*, protease structure with cocrystallized natural peptide substrate (in *orange*) (from Protein Data Bank [PDB] ID: 3M5O (62)); the protein backbone given as *ribbon* model with transparent surface depiction. Residues of interest highlighted as *stick* models and colored as follows: residue 80, *blue*; residue 91, *light green*; and residue 174, *dark green*. *B*, impact of NS3-Q80K and epistatic secondary substitutions on the protease protein flexibility estimated by RMSFs over time for different NS3-Q80K mutant patterns. *C*, protease protein flexibility averaged over time for protease WT (from PDB ID: 1CU1 (48)) and NS3-Q80K mutants represented as tubes. Epistatic secondary substitutions differentially affect the bilobal fold of the protease. We used GROMACS to generate RMSF B-factors added to the PDB file. We then used PyMOL defaults to visualize flexible, stable, and intermediate residues. Highly flexible residues are shown in *red*, highly stable residues are shown in *blue*, and the intermediate flexible residues are shown in *white*. The size of the tubes is proportional to the observed RMSF values in those regions.

The Q80 residue resides in the protease–helicase domain interface of NS3 close to the protease active site (Fig. 5*A*). The epistatic site S174 resides in close proximity to Q80 (approximately 3.9 Å distance), exhibiting hydrogen bonds between both residues. In contrast, the second epistatic site A91 is more than 20 Å distant from residue Q80 (Fig. 5*A*). For simulations, we created protease mutant structures corresponding to NS3-Q80K with or without epistatic amino acid substitutions (equivalent to mutants from Table 1).

During molecular dynamics (MD) simulation, we observe destabilization of the protease structure upon NS3-Q80K mutation in the N-terminal lobe of the protease (approximately residues 7–78). Destabilization from NS3-Q80K in part is alleviated by the epistatic secondary substitutions S174N and/or A91S/T. The modeling results are in line with the particular protein denaturation curves that we observed for NS3-Q80K and epistatic secondary substitutions (Fig. 5, *B*–*F*). They indirectly validate our hypothesis on the differential impact of mutations on the bilobal structure of the protease. Particularly, mutant patterns comprising S174N or A91T showed fold-stabilizing potential, as witnessed by root mean square fluctuation values for each trajectory (Fig. 5*B*). We have quantified this observation by extracting 181 frames from the concatenated MD trajectory and computing the number of contacts for each of the amino acid substitutions at positions 80, 91, and 174 (Fig. 5 and Fig. S1). We observe that while the number of contacts of individual positions fluctuates, and in particular, the number of contacts of position 80 decreases upon Q80K mutation, the total number of contacts agrees well with the stability measurements (Fig. 3) and the root mean square fluctuation values from the MD trajectories. Interestingly, new contacts and hydrogen bonds of the mutated amino acid at position 91 contribute most to this trend. This finding agrees with previous evidence that long-range contacts generally make greater thermodynamic impact than contacts that are near in sequence and structure (28).

### Epistatic secondary substitutions impair the replicative fitness of NS3-Q80K variants

**Figure 6:**
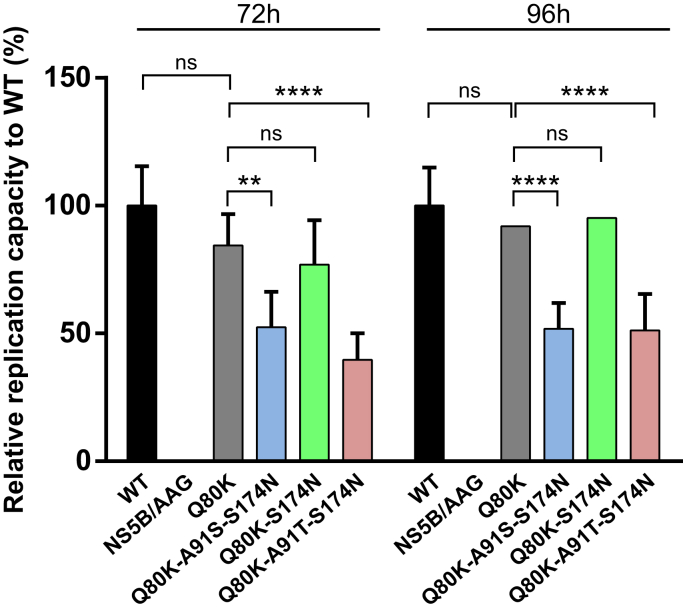
**Impact of epistatic amino acid substitutions on RNA replication of NS3-Q80K variants.** Medium was collected and replaced at 8, 24, 48, 72, and 96 h after transfection of H77S.3/GLuc2A RNAs carrying the indicated mutations, and GLuc activity determined at 72 h (*left panel*) and 96 h in time (*right panel*). Results normalized to the 8 h GLuc activity represent the mean of triplicate samples and are representative of multiple experiments. Error bars represent the mean ± SD from at least three independent experiments; ∗*p* ≤ 0.05; ∗∗*p* ≤ 0.01; ∗∗∗*p* ≤ 0.001; and ∗∗∗∗*p* ≤ 0.0001; by two-way ANOVA. GLuc, Gaussia luciferase.

Based on the biochemistry data and MD simulations (see previously), we speculated that epistatic interactions in NS3-Q80K by stabilizing the protease protein fold could support the replicative fitness of viral variants by mechanisms related to the protease–helicase domain interplay. To assess the replication capacity of NS3-Q80K ± epistatic secondary substitutions, the amino acid substitutions were created within the background of the genotype 1a H77S.3 genome, and their impact on replication of the viral RNA was determined in RNA-transfected cells. H77S.3/Gaussia luciferase (GLuc)2A RNA was assessed from NS3-Q80 protease WT and the NS3-Q80K protease ± epistatic secondary substitutions by measuring GLuc activity in supernatant media collected at 24-h intervals after transfection of synthetic RNA, as described previously. Results were normalized to the GLuc activity present at 8 h after transfection, as this represents GLuc expressed directly by the transfected input RNA. An H77S.3 virus comprising a protease with Q80-A91-S174 sequence pattern is denoted WT virus. This virus was able to replicate efficiently in the cell culture model of infection (Fig. 6).

We find that the Q80K polymorphism in the H77S.3 backbone showed only minimal loss of replication capacity at 72 h after transfection compared with NS3-Q80 protease WT while it reached WT level at 96 h. Unexpectedly, the mutant patterns from our study did not increase the replicative fitness of NS3-Q80K variants. We find the GLuc activity of the triple mutant Q80K-A91T-S174N at 72 and 96 h close to 50% or lower compared with the WT virus (48.8% decrease compared with WT at 96 h; FC = 0.51). In contrast, the GLuc activity of the double mutant Q80K-S714N was only moderately impaired at 72 h (23.1% decrease compared with WT; FC = 0.77) and was not significantly different at 96 h (4.8% decrease compared with WT; FC = 0.95). However, none of the epistatic amino acid substitutions demonstrated enhanced RNA replication capacity compared with the parental H77S.3/GLuc2A RNA or NS3-Q80K.

## Discussion

Fitness-compensatory mutations are pivotal drivers of viral quasispecies evolution, but structural constraints restrict their evolutionary space (29, 30, 31). We previously reported the delicately balanced protease–peptide interactions in NS3-4A that limit the protease adaptive capability to preclude peptide substrate overfitting and competitive enzyme inhibition (32). As a consequence, secondary substitutions to rescue fitness deficits are observed only rarely in NS3-4A. A study by McCloskey *et al.* (1), however, identified amino acid substitutions in NS3-4A that epistatically “interact” with NS3-Q80K and obviously contribute to the phylogenetic stability of the polymorphism in viral variants. The molecular mechanisms underlying epistatic interactions in NS3-Q80K likely differ from previously observed fitness-compensatory mechanisms in PI-resistance mutations (18, 32, 33, 34, 35). Albeit clinically important in treatment failure with DAA agents, they so far remained unexplained.

One of the constraints on fast-evolving viruses, such as hepatitis C, and the evolution of proteins is the stability of the folded “native” protein state (36). Essentially, the epistatic amino acid substitutions from our study stabilized the NS3-Q80K protein fold and leveraged the WT protease level. By MD simulations, we find changes in the total number of contacts between protease residues in NS3-Q80K and epistatic amino acid substitutions that reflect destabilizing *versus* stabilizing effects on the protease protein structure. This is in agreement with previous findings from Peres-da-Silva *et al.* (37) reporting variations in hydrogen bond occupancies from epistatic interactions in NS3-Q80K. The total number of contacts in our structural models agreed well with our protein stability measurements. This reflects the compensatory effect on the protein stability from epistatic amino acid substitutions in NS3-Q80K. Interestingly, the bioinformatics analysis shows that Q80K mutation destabilizes the protein by affecting the N-terminal subdomain of the protease fold. The shape of the experimental temperature stability curves can reflect the differential stability of different domains of a protein, with increased formation of a shoulder when the *T*_m_s between two domains are more distant between each other (38). We suggest that, by differentially affecting the stability of the two subdomains of the protease fold, the mutations change the shape of the denaturation curves: the start of the denaturation shifts to lower temperatures for the N-terminal domain of the protease because of destabilization by Q80K with a corresponding small shoulder around 45 °C, unmasking the bilobal protease fold. A more pronounced change in the shape of the denaturation curve is then observed for Q80K with additional mutations at A91 and S174N that reflect the Q80K early denaturation with the extended stability to higher temperatures because of epistatic substitutions. However, thermodynamic stability is subject to various conflicting forces that trade-off against each other, in particular protein function and specificity, which illustrates the complexity of the evolutionary forces (36, 39). Accordingly, we observed a trade-off in protease catalytic activity from increased protein stability in NS3-Q80K harboring epistatic secondary substitutions that also limited the replication fitness of the respective variants. Based on our data, however, it seems unlikely that replicative fitness is a limiting factor in the selection of NS3-Q80K variants in virus evolution. We find no significant deficit in the replication fitness of NS3-Q80K variants that require rescue by secondary substitutions. Murai *et al.* (40) observed a replication boost of Q80K-R155K variants in a genotype 1a infectious cell culture model of infection. However, by using purified protein in our protease activity assay, we find that none of the epistatic secondary substitutions could even leverage the NS3-Q80K protease functional level, but some nearly abolished peptide substrate turnover. Nevertheless, epistatic secondary substitutions that most effectively compensate protein stability select under DAA pressure in treatment-failure patients harboring NS3-Q80K (Fig. 1). This is in contrast to the prevalence of Q80K-A91S with only minor fold-stabilizing potential (Figs. 1 and 3 and Table S1) that decreased upon DAA failure.

Notably, NS3 has both protease and RNA helicase enzymatic activities that modulate their respective functions and play important roles in the propagation of HCV (19, 20). We considered the possibility that stabilizing the protease protein fold could potentially support domain–domain interactions and thereby contribute to the phylogenetic stability of the Q80K polymorphism. Our protease functional assay, however, lacks the NS3 helicase domain (27) and cannot capture mechanisms related to the domain interplay. However, we could investigate the effect of the mutations on the full-length protein in a cellular model of viral replication that requires the helicase functionality. Hence, we assessed related mechanisms of epistatic amino acid substitutions in NS3-Q80K in an infectious cell culture model. Surprisingly, only Q80K-S174N in cell culture showed replicative fitness that equals the NS3-Q80K level. Yet, none of the epistatic amino acid substitutions boosted the replicative fitness of NS3-Q80K variants. We reasoned that attaining increased stability by epistatic residue interactions likely impact the NS3-Q80K protease ability to adopt novel functions not directly related to the virus replication cycle.

**Figure 7:**
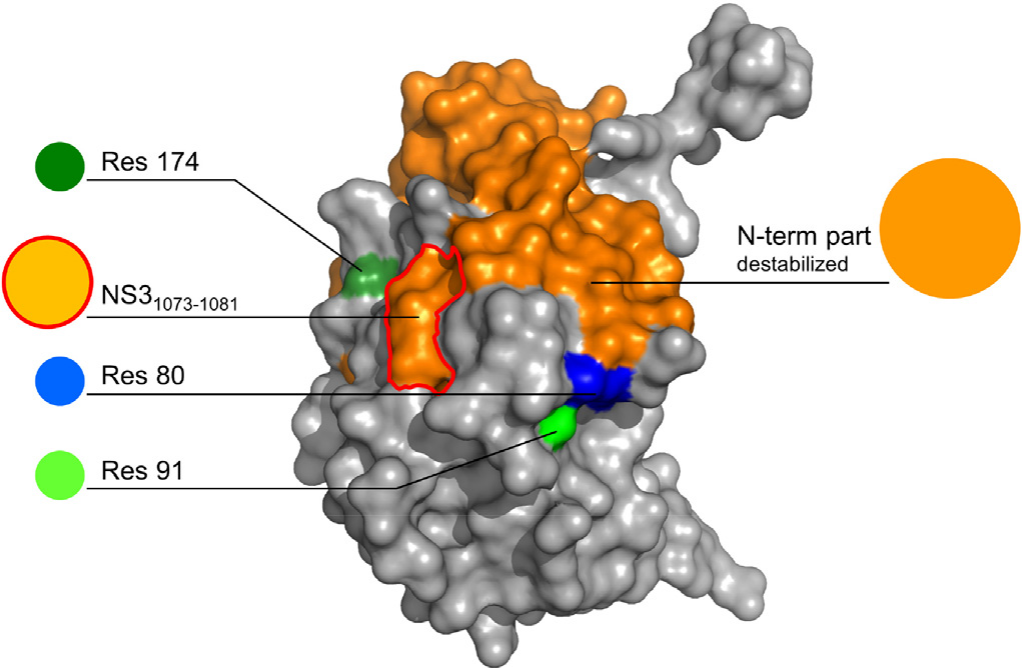
**Localization of NS3-Q80K, epistatic interaction sites, and the T-cell epitope NS3**_**1073–1081**_**.** Surface representation showing the destabilized N-terminal part of the NS3-Q80K protease structure (*orange*; residues 7–78, based on RMSF from Fig. 5*B*) (from Protein Data Bank ID: 3M5O (62)) comprising the immunodominant T-cell epitope NS3_1073–1081_ (*red circled*; corresponding to protease residues 47–55); residue 80 (*blue*); and epistatic interaction site 91 (*light green*) and 174 (*dark green*). RMSF, root mean square fluctuation.

As shown previously, the NS3-4A protease function not only plays an important role for escape from immune pressure (18, 32) but also is a prime epitope for cytotoxic T lymphocyte (CTL)–mediated immune responses (41). T-cell responses against the human leucocyte antigen class I–restricted immunodominant protease epitope NS3_1073–1081_ have frequently been associated with clearance of acute HCV infection (42). Several studies have confirmed the immunodominant role of this epitope in human leucocyte antigen-A2+ patients and provided evidence for a role of CTLs to this epitope in the control of infection (41, 42). To escape CTL recognition and promote viral persistence, the virus can mutate within immunodominant epitopes. Although such mutations can completely abolish recognition by CTLs, variability within the T-cell receptor–binding domain is accompanied by severe viral variant fitness deficits that limit their selection in nature (42). The two epistatic NS3-Q80K interaction sites A91 and S174, however, are located outside the NS3_1073–1081_ epitope (Fig. 7). Accordingly, the corresponding epistatic substitutions, in our cell culture model, only had a minor impact on the replicative fitness of NS3-Q80K variants, although they showed significant potential to rescue protease fold instability including the NS3_1073–1081_ epitope. Interestingly, fold-stabilizing mutations can mediate escape from antiviral immunity, as previously reported (43). The underlying mechanism depends on the conformational stability of the target peptides. Protein unfolding is rendering the peptide backbone accessible to proteolytic degradation, thereby determining the availability of appropriate antigenic peptides that can trigger CTL responses (44, 45). To our knowledge, thermal stability so far is unknown as an immune escape mechanism in HCV. A prominent example, however, is reported from the study by Klein *et al.* (43) in influenza A virus infection. They found that pandemic H1N1 influenza A strains split into two lineages that had different relative hemagglutinin protein stabilities with later variants descended from the higher-stability lineage. Analysis of the mutations that constrained the influenza A virus evolution associated with selective sweep showed early appearance of highly stabilizing mutations in the hemagglutinin protein (43). Similarly, CTL immune escape in HCV could explain the stable transmission of NS3-Q80K between hosts. Such a mechanism would also support the replicative fitness of NS3-Q80K variants in the presence of DAAs and could contribute to a general drug resistance phenotype (46) (Fig. 1).

In summary, our results suggest that protease protein stability plays a significant role in the persistence of NS3-Q80K variants. Analysis of the amino acid substitutions associated with NS3-Q80K found that they were characterized by rescue in protein stability that trade-off against protease function. The data lead us to conclude that epistatic interactions in NS3-Q80K contribute to the high prevalence of this polymorphism by mechanisms not directly related to RNA replication. By stabilizing part of the protein structure comprising a prime epitope for CTL-mediated immune responses, epistatic interactions potentially protect NS3-Q80K variants from detection by T cells.

## Experimental procedures

### Study populations, sequence retrieval, and analysis

Sequence analysis for secondary substitutions in the NS3-4A protease of genotype 1a with NS3-Q80K was based on sequence information deposited in the Frankfurt Resistance Database from serum of HCV patients with DAA failure sent from European study sites. At least 73% of these patients received first-line DAA therapy. Twenty-four patients (58.5%) were treated with paritaprevir/ritonavir—ombitasvir and dasabuvir, the 3D regimen (PrOD), eight patients (19.5%) with simeprevir/sofosbuvir, and nine patients (22%) with grazoprevir/elbasvir (Table S1). The standard treatment duration was 12 weeks. The majority of patients experienced relapse (81%), whereas a nonresponse or breakthrough was detected in only approximately 10% of individuals (the remaining 9% of patients discontinued treatment, or the type of failure was not specified). Sequence information on DAA-naïve patients of genotype 1a harboring NS3-Q80K was retrieved from the European HCV database (24). Details are provided in the supporting information.

### Protein biochemistry

The protein construct used for thermal shift and protease activity assays comprises the N-terminal protease domain of NS3 linked to the NS4A peptide of the viral polyprotein. For transformation and recombinant NS3-4A protein expression (27), the pET-15b vector incorporating the single-chain proteolytic domain (NS4A_21–32_–GSGS-NS3_3–181_) of the NS3-4A protease WT or mutants of NS3-4A was transformed into *Escherichia coli* Rosetta pLysS (DE3) competent cells (Novagen) according to the manufacturer's protocol. Recombinant protein production and purification was carried out as described previously (32).

#### Thermal shift assay

Stability of the protein fold of expressed WT and mutant NS3-4A was measured by fluorescence-based thermal shift using Sypro Orange, a temperature-stable fluorophore that exhibits enhanced fluorescence upon interacting with unfolded proteins (25, 32). The impact of NS3-Q80K and epistatic amino acid substitutions at protease residue A91 and/or S174 (in double and triple mutant patterns) on protease protein unfolding patterns was characterized by fluorescence emission curves. *T*_m_ from purified protease WT and mutants is determined by fitting the sigmoidal melt curve to the Boltzmann equation.

#### Protease activity assay

The enzymatic activity and Michaelis–Menten kinetics of the NS3-4A protease WT and mutant proteases were measured by a fluorescence-based enzyme activity assay (26, 32). To measure peptide cleavage kinetics, a viral polyprotein substrate derived from the NS4A/4B cleavage site was purchased from Anaspec (SensoLyte 520 HCV; Anaspec).

### Computational studies

The models for the NS3-Q80K protease mutants were created with FoldX (http://foldxsuite.crg.eu) (47) based on the structure of the NS3 protease–helicase complex from genotype 1b (48) (Protein Data Bank ID: 1CU1), which in the protease domain is 92% identical to the genotype 1a enzyme. A structure of the protein monomer was extracted for further analysis. The structures were optimized, and MD simulations were performed with GROMACS, version 5.1.4 (https://www.gromacs.org) (49), using Amber99SB∗-ILDN force field (50, 51). The molecules were centered in a cubic box with a 1.5 nm buffer, under periodic boundary conditions, and the systems were explicitly solvated with TIP3P water molecules. Counterions (52) ClJ and NaJ for Amber99SB∗-ILDN force field simulations were added when necessary to neutralize the overall charge (0.15 mol/l concentration). Energy minimization for each of the molecular systems was performed using the steepest descent algorithm. A maximum of 50,000 steps was performed until a maximum force of 1000.0 kJ mol^−1^ nm^−1^ was achieved. Following the energy minimization, each of the molecular systems was subject to two consecutive steps of equilibration procedure. At first, each system was maintained at a temperature of 310 K during the canonical (NVT; particle number N, volume V and temperature T) ensemble for 100 ps with a time step of 2 fs, followed by a 100 ps simulation in the isothermal–isobaric (NPT; particle number N, pressure P and temperature T) ensemble with a time step of 2 fs maintaining the pressure at 1 bar to equilibrate the system.

After the NPT ensemble simulations, we performed 28 production simulations with 100 ns each and four replicas for each of the NS3-Q80K models (WT protease and NS3-Q80K mutants). The coordinates were recorded at every 100 ps. The temperatures of solute and solvent were coupled separately to the velocity rescale thermostat (modified Berendsen thermostat) (53) at 310 K with a relaxation time of 0.1 ps. The pressure was maintained at 1 atm by isotropic coordinate scaling with a relaxation time of 5 ps using Parrinello–Rahman barostat (54). A time step of 2 fs was used to integrate the equations of motion based on the leap-frog algorithm (55). Lennard–Jones interactions were set to a cutoff of 1.4 nm, and the Particle Mesh Ewald (56) method was used to treat long-range electrostatic interactions. All bonds were constrained using P-LINCS algorithm (57).

From the resulting trajectories, the first 10 ns were removed as part of the equilibration process, and the rest of the 90 ns from each of the simulated protease models were concatenated with its own replicas. From each concatenated trajectory, 181 frames were extracted at equal time steps, and residue interaction networks were created using RINerator (https://rinalyzer.de/rinerator.php) (58). The number of contacts for each position of interest was investigated with custom scripts in Python.

### Infectious HCV cell culture

Details of the cells and reagents used for characterization of viral variant replicative fitness in H77S cell culture are reported elsewhere (23).

#### Plasmids

The pH77S.3 and pH77S.3/(GLuc)2A are molecular clones of the genotype 1a H77 strain of HCV (59). The H77 strain harbors the sequence pattern Q80-S91-N174, which differs from the consensus sequence pattern Q80-A91-S174 in HCV genotype 1a–infected individuals (60). Epistatic amino acid substitutions, A91S/T and S174N, were created by site-directed mutagenesis in plasmids harboring NS3-Q80K. pH77S.3/GLuc2A RNA produces secreted GLuc reporter protein.

#### Virus fitness

Genome-length RNA was transcribed from the mutated pH77S.3 and pH77S.3/GLuc2A plasmids *in vitro*, and studies to assess viral fitness were carried out as described previously (23).

### Statistical analyses

Comparisons of measurements from thermal shift and protease activity assay were analyzed by two-sided *t* tests with a level of significance set at a minimum of *p* = 0.05. Two-way ANOVA was used to compare the magnitude of changes in RNA replication after normalization to WT controls. All statistical tests were carried out using Prism 6.0 software (GraphPad Software, Inc).

## Data availability

All data that support the findings of this study are available from the corresponding author upon request.

## Supporting information

This article contains supporting information (61).

## Conflict of interest

The authors declare that they have no conflicts of interest with the contents of this article.
