# Supplementary material for: Epistatic interactions promote persistence of NS3-Q80K in HCV infection by compensating for protein folding instability
Source: J Biol Chem. 2021 Jul 31;297(3):101031. doi: 10.1016/j.jbc.2021.101031 (PMC8405986; doi:10.1016/j.jbc.2021.101031)
Supplement: Supplemental Figure S1 and Table S1 [file mmc1.docx]

**SUPPORTING INFORMATION**

**Epistatic interactions promote persistence of NS3-Q80K in HCV infection by compensating for protein folding instability**

Georg Dultz^1¶^, Sanjay K. Srikakulam^2,3,4¶^, Michael Konetschnik^1¶^, Tetsuro Shimakami^5^, Nadezhda T. Doncheva^6^, Julia Dietz^1^, Christoph Sarrazin^1§^, Ricardo M. Biondi^7^, Stefan Zeuzem^1,8^, Robert Tampé^9^, Olga V. Kalinina^2,10,11^, Christoph Welsch^1,8^*

**SUPPLEMENTARY MATERIALS AND METHODS.**

**Study populations, sequence retrieval and analysis.** Based on serum of HCV patients with DAA failure sent from European study sites to our laboratory for routine diagnostic HCV resistance testing, we analyzed sequence data of genotype 1a with NS3-Q80K deposited in the Frankfurt Resistance Database. Information of the pretreatment status was provided on a resistance analysis submission form, including the documentation of limited parameters, such as cirrhosis, pretreatment status, administered DAA regimen, treatment duration and virologic response. Patients who received at least 4 weeks of antiviral treatment were included in this study. Investigations were performed according to the Declaration of Helsinki, and approval of the usage of patient blood samples and retrospective collection of data for research purpose was obtained from the local ethics committee (Ethikkommission der J. W. Goethe-Universität Frankfurt).

Sequence information on DAA-naïve patients of genotype 1a harboring NS3-Q80K was retrieved from the European HCV database^24^ (https://euhcvdb.lyon.inserm.fr/euHCVdb/). Sequence information was downloaded on April 23, 2014 after filtering based on the following criteria: single protein, standard name ns3, confirmed subtype and published before 2010. The majority of these sequences could be confirmed as derived from patients who had not been treated or who were undergoing interferon therapy. None were reported to have received DAA therapy. For those sequences for which there was no information regarding prior therapy, the year in which the data were deposited and the source of the sequence (none were deposited by pharmaceutical companies) preclude prior participation of the patient in a clinical trial of a DAA.

We computed multiple sequence alignments with minor manual modifications using the SEAVIEW alignment editor^61^.

**SUPPLEMENTARY REFERENCES.**

1. Gouy M, Guindon S, and Gascuel O. (2010) SeaView version 4: a multiplatform graphical user interface for sequence alignment and phylogenetic tree building. *Molecular Biology and Evolution* **27**, 221-224.

**SUPPLEMENTARY TABLES.**

**Table S1. Epistatic secondary substitutions in DAA-naïve and DAA-experienced patients harboring NS3-Q80K.**

| **Variant** | **DAA naïve^#^** n (%) | **DAA**  **failure^§^** n (%) | **Regimen^§^** | | |
| --- | --- | --- | --- | --- | --- |
|  |  |  | **SMV/SOF** n (%) | **3D (PrOD)** n (%) | **GZR/EBR** n (%) |
| Q80K | 5 (5) | 5 (12) | 0 (0) | 4 (17) | 1 (11) |
| Q80K-A91S | 13 (13) | 1 (2) | 0 (0) | 0 (0) | 1 (11) |
| Q80K-A91T | 1 (1) | 1 (2) | 0 (0) | 0 (0) | 1 (11) |
| Q80K-S174N | 37 (37) | 10 (24) | 2 (25) | 4 (17) | 4 (44) |
| Q80K-A91S-S174N | 22 (22) | 11 (27) | 4 (50) | 7 (29) | 0 (0) |
| Q80K-A91T-S174N | 21 (21) | 13 (32) | 2 (25) | 9 (38) | 2 (22) |
| other | 1 (1) | 0 (0) | 0 (0) | 0 (0) | 0 (0) |

**Table legend:** Patients with genotype 1a HCV infection harboring NS3-Q80K from the European HCV database^24^, euHCVdb (DAA naïve, n=100)^#^ and patients with NS3-Q80K from the Frankfurt Resistance Database^§^ upon treatment failure (DAA failure, n=41); DAA regimens: 3D, paritaprevir/ritonavir, ombitasvir plus dasabuvir (PrOD) (n=24); GZR/EBR, grazoprevir plus elbasvir (n=9); SMV/SOF, simeprevir plus sofosbuvir (n=8). Data shown represent patients from which the respective variants were isolated as major variants before DAA treatment or upon treatment failure.

**SUPPLEMENTARY FIGURES.**

**
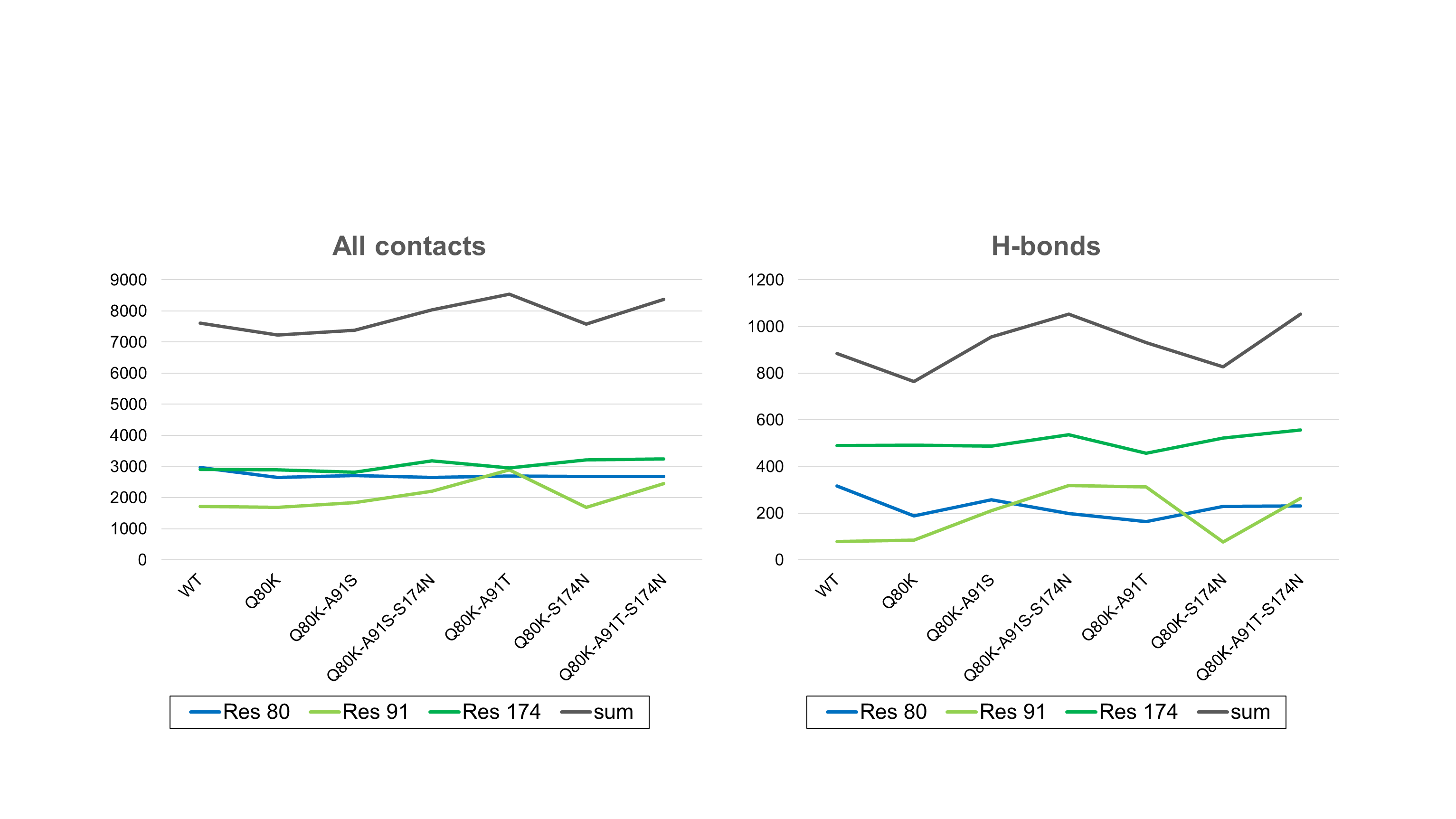
Figure S1.**

**Number of contacts quantified from concatenated MD trajectories.** Total number of contacts (left) and number of hydrogen bonds (right) in equally spaced 181 frames from the MD trajectories.
